# Synthesis, Morphologies and Building Applications of Nanostructured Polymers

**DOI:** 10.3390/polym9100506

**Published:** 2017-10-13

**Authors:** Yong Lu, Kwok Wei Shah, Jianwei Xu

**Affiliations:** 1 School of Design and Environment, National University of Singapore, 4 Architecture Drive, Singapore 117566, Singapore; luyong9720@gmail.com; 2 Institute of Materials Research and Engineering (IMRE), Agency for Science, Technology and Research (A*STAR), 2 Fusionopolis Way, Singapore 138634, Singapore

**Keywords:** nanostructured polymers, polymer nanoparticles, polymer nanofibers, building applications

## Abstract

Nanostructured polymers (NSPs) are polymeric materials in the size of nanoscale, normally consisting of nanoparticles, nanofibers, nanowires, nanospheres and other morphologies. Polymer nanoparticles (PNPs) can be fabricated either by physical methods (i.e., solvent evaporation, nanoprecipitation, salting out) or by direct nanosynthesis, using micro- or nanoemulsions with nanoreactor compartments to perform polymerization. Polymer nanofibers (PNFs) can be produced via various techniques and the most commonly used approach is electrospinning, whereby a charged solution of a polymer when exposed to an opposite high electric field is pulled into long thin nanofibers. NSPs in general exhibit enhanced properties such as excellent structural and mechanical properties, making them promising candidates for some particular building applications. A variety of PNFs have been developed and used for noise and air pollution filtration. Moreover, PNFs can also be fabricated with phase change materials which are usually employed for thermal energy storage in construction industry. In this review, we will summarize the morphologies and nanosynthesis methods of NSPs, in particular, PNPs and PNFs. In addition, representative NSPs mainly used in construction are introduced for building applications.

## 1. Introduction

Polymers are one of the most important engineering materials [1,2]. Over the last four decades, the usage of polymeric materials in construction has grown rapidly mainly due to the following factors: (1) The availability of basic raw materials for massive production; (2) The outstanding properties, for example, light weight, chemical stability and elasticity, etc.; (3) Easy and flexible processing methods; (4) Benefits in economy, for example, raw materials and manufacturing cost, maintenance and operational cost, etc.; (5) Reduction of environmental cost. As such, polymers, together with cement, ceramics, woods and metals mainly including aluminium, copper and steel, represent the essential materials in the construction industry [3,4,5].

Several key properties are to be considered if a polymer is suitable for construction applications. First of all, its mechanical properties are of the highest importance for all applications in construction. Polymer’s mechanical properties can be determined by testing its deformation and flow characteristics under stress, and such a polymer can subsequently be classified to use as an elastomer (rubber-like products), rigid or flexible plastics, or as a fiber depending on its measured mechanical behavior obtained. Secondly, thermal properties of a polymer usually refer to its thermal stability defined by the temperature range over which it can maintain its useful properties. Commonly, polymers have low thermal conductivity and thus the materials have found application as insulators. Thirdly, polymer flammability is another important factor to be considered in construction. Usually, the response of construction polymeric materials to a combustion process is very complicated and depends on the specific type and detailed additives used in the polymers. For example, the engineering thermoplastics tend to soften and flow before ignition takes place whereas the thermosetting plastics rather undergo surface charring and sometimes the charred residue even forms an insulating layer of the flaming place. Other properties such as weathering, permeability, chemical stability, etc., are important as well and they can greatly affect the polymer performances. Comprehensive consideration and evaluation must be made before applying a polymer for real building applications. Several polymers and their common applications in construction are listed in Table 1.

The rapid development in the field of nanotechnology allows for new opportunities in the application of nanostructured polymers (NSPs) as construction materials. Polymer nanotechnologies not only enhance material properties and other unique functions but also facilitate energy conservation [6,7,8]. NSPs have been prepared via various techniques and their applications for buildings have been investigated. Among NSPs such as polymer nano-particles, -fibers, -wires, -spheres, etc., polymer nanoparticles and nanofibers are most widely investigated and applied [9,10,11,12]. Herein, the following section will give a brief introduction to the synthetic methods of polymer nanoparticles and nanofibers.

### 1.1. Polymer Nanoparticles (PNPs)

Nanoparticles (NPs) are defined as solid, colloidal particles in at least one dimension of 1–1000 nm. Thus, polymer nanoparticles (PNPs) are referred to polymeric particles which are in nanosize, typically for nanospheres and nanocapsules. Nanospheres are solid particles that have entire solid mass spherical shape. Nanocapsules are core-shell structure particles. Generally, a liquid core (either oil or water) is surrounded by a solid polymeric shell which is a kind of protection strategy for core materials to surroundings [13,14,15].

The methods for synthesis of PNPs can be generally divided into physical and chemical approaches, for example, dispersion and polymerization respectively. Dispersion strategy includes solvent evaporation/extraction [16,17], nanoprecipitation [18,19], salting-out [20,21], dialysis [22], etc. while polymerization strategy includes emulsion polymerization [23,24], mini/micro emulsion polymerization [25,26], interfacial polymerization [27,28], etc. Among these preparation techniques, solvent evaporation/extraction, nanoprecipitation, salting-out and emulsion polymerization are mostly used for fabrication of PNPs.

Solvent evaporation/extraction was the first method to prepare PNPs from a preformed polymer [29]. A typical procedure involves a synthetic or natural polymer dissolved in volatile solvent, usually chloroform, dichloromethane and ethyl acetate while water is present with stabilizer to form an emulsion system. Then solvent is gradually removed via evaporation either by continuous stirring at room temperature or reduced pressure. Upon evaporation of the solvent of the polymer, a nanoparticle suspension, which is allowed to diffuse through the continuous phase of the emulsion, is consequently formed. The solidified nanoparticles can then be collected by ultracentrifugation and washed with distilled water to remove additives such as surfactants. Although solvent evaporation is the widely employed technique to prepare PNPs, usually high energy is required for homogenization, making the application challenging and impractical on a pilot scale [30].

The nanoprecipitation method of preparing PNPs is also called as solvent displacement method [31]. It is based on drop-wise addition of organic phase (polymer dissolved in water-miscible solvent, i.e., acetone, ethanol, and THF) into an aqueous phase. The nanoprecipitation system consists of three basic components: the synthetic or natural polymer, low boiling point miscible solvent and immiscible solvent (usually water). Thus, acetone is the most frequently employed polymer miscible solvent in this method. Upon addition of polymer solution into its immiscible solution, rapid diffusion of the solvent into non-solvent phase results in the decrease of interfacial tension between the two phases, increasing the surface area and subsequently leading to the formation of small droplets of organic solvent. PNPs herein could be obtained with the removal of organic solvent. The main advantages of nanoprecipitation approach to synthesize PNPs are high encapsulation efficiency, narrow size distribution, and ease of scale-up.

Salting-out method is a modified emulsification process which is achieved by adding a high concentration of salt, avoiding the usage of surfactant and chlorinated solvents [32]. Commonly, electrolyte, i.e., magnesium chloride, calcium chloride and magnesium acetate or non-electrolyte i.e., sucrose are used as add-in salt. The typical procedure involves the formation of polymer emulsion, which is achieved by mixing a polymer solution with a concentrated salt solution. Then the polymer emulsion is diluted with a large excess of water, leading to the precipitation of the polymer from emulsion droplets due to the solvent migration. Extensive washing of the prepared nanoparticles is mandatory for salting-out technique.

The PNPs prepared using techniques mentioned above are from preformed polymers and do not involve any polymerization processes. Therefore, these techniques limit the preparation of PNPs in a wide variety. Direct synthesis is a possible way to make objects in nanosize, so preparation of PNPs can be done via the polymerization of monomers by careful design. Although suspension, interfacial and living radical polymerization can be employed in the preparation of PNPs, emulsion polymerization is the most common method used for the production of PNPs. The benefits of such a polymerization include (1) the use of water as the dispersion medium which is environmentally friendly; (2) excellent heat dissipation during the course of the polymerization; (3) rapid polymerization to yield high molecular weight polymers with low polydispersity; (4) easy separation of final product; and (5) mild and cost-efficient reacting conditions [33]. In a typical emulsion system, the ingredients comprise water, a monomer with low water solubility, initiator and a surfactant. Polymerization initiates when a monomer molecule collides with an initiator molecule which could be an ion or a free radical. Alternatively, the monomer molecule can also be transformed into an initiating radical by radiation, ultraviolet or strong visible light. Phase separation and formation of solid particles can take place as polymerization reaction goes on. The final solid PNPs can be easily separated from an aqueous system and collected by filtration [34].

### 1.2. Polymer Nanofibers (PNFs)

Polymer nanofbers (PNFs) are defined as polymer based fibers that are at least one dimension in the size of 100 nm or less. Compared with common bulk, PNFs demonstrate comparable size-dependent behaviors similarly to other nano-objects. Experimental studies have demonstrated that PNFs will have a set of favorable properties such as increased surface-to-volume ratio, controlled release of drugs, high anisotropic electrical conductivity, enhanced light scattering and photoluminescence as well as outstanding mechanical and thermodynamic properties [35]. As a new class of promising materials, PNFs have been developed for many applications including drug delivery, filtration, wipes, barriers, garments, composite, and insulation, etc. [36].

There are a number of methods that are used for synthesis of PNFs in recent years such as drawing, template synthesis, phase separation, self-assembly and electrospinning. Among these techniques, electrospinning process seems to be the only approach that is for massive production and can be used for production of one-by-one continuous PNFs whereas other techniques are used to fabricate PNFs in small scales.

Drawing process of PNF preparation involves a micropipette or a glass rod in contact with polymer solution droplet which is then withdrawn slowly subsequently [37]. Single solid PNF is thus produced due to solvent evaporation as the micropipette or glass rod is moving. The drawing technique can make very long single NFs but as-prepared polymer solution should have certain viscoelastic properties for proper drawing. On the other hand, as the solvent evaporates from the deposited polymer droplets, the viscosity of the system continuously increases, so the time of drawing is undergone limitation, resulting in a specific diameter of the prepared PNFs.

Template synthesis of PNFs uses membranes or templates to make NFs of solid or hollow shape structure [38]. Nanoporous raw material membranes based on electronically conducting polymers, metals/metal oxides, semiconductors, and carbons can be used as a template for fabrication of PNFs. Among the templates, metal oxide membranes (e.g., aluminum oxide membrane) are commonly used. Typically, a polymer solution passes through the membranes to a non-solvent bath to produce PNFs depending on the pore diameter. However, the method can hardly prepare continuous NFs one-by-one.

Phase separation synthesis of PNFs is usually used to prepare three-dimensionalobjects [39]. The procedure consists of dissolution, gelation, extraction using a different solvent and then freezing, drying to form a nanoscale porous foam. Typically, a non-solvent is added to a polymer solution to produce a polymer-rich phase and a solvent-rich phase. The polymer-rich phase is subsequently fixed by quenching under low temperature, followed by the solvent removal via freeze-drying or extraction, thereby producing porous 3-D polymer nano-object. Phase separation synthesis is a simple technique that does not require much specialized equipment and is easy to achieve batch-to-batch consistency. However, only a selected number of polymers can be used for the method and the process takes relatively long period.

Self-assembly synthesis of PNFs is a process in which individual units organize themselves into defined patterns to form nanostructured geometries [40]. Usually, small individual components are spontaneously organized through intermolecular interaction into an ordered and stable structure. Such a self-assembly behaviour of synthetic or natural macromolecules thus can produce supramolecular structures in nano/micro scale and thus NFs can be obtained as well. Self-assembly synthesis of PNFs can produce very thin fibers which are in the diameter of several nanometers. However, the technique is time-consuming and is hardly used for mass production.

Electrospinning synthesis of PNFs is a simple and versatile process and most widely used for the production of PNFs [41,42,43]. Additionally, the technology can be applied to most polymer materials. In general, an electrospinning system consists of three components: a capillary tube with a pipette or needle of a small diameter, a metal screen for collecting, and a high-voltage source for generating electric field. Typically, a polymer solution is introduced between two electrodes which generate the electric field. One of the electrodes is placed into the polymer solution and the other is attached to the metal collector. Polymer filaments are then formed in solution between the two electrodes under high voltage. Once ejected out of the pipette or needle, the charged polymer solution jets evaporates to become fibers and are collected on the metal collector. Parameters such as polymer molecular weight, solution viscosity, pipette/needle diameter and distance between can be tuned to generate fibers of various thicknesses, from several nanometers to micrometers. However, if the distance between the needles and the collectors is short, PNFs tend to stick to the collecting device as well as to each other because of incomplete solvent evaporation. It is interesting to note that all sorts of PNFs, including single fibers, composite fibers, core–shell structure fibers and porous or hollow fibers, can be generated by electrospinning. Moreover, massive production of PNFs can be achieved by this technique or by parallel arrangement. Several applications have been developed for those fibers, i.e., filters, membranes, biodegradable scaffolds, drug release agents, or fillers in nanocomposites.

Although many of the published work have reviewed the synthesis and morphology of PNPs and PNFs, review on the polymers commonly used in construction for their nanostructures remains scarce. In this review, we focus on two areas closely related to these NSPs, (1) synthesis and morphology (2) building applications. Our intention is to fill in missing gaps and give readers a general understanding of NSPs from their synthesis, morphology and building applications.

## 2. Nanosynthesis of Representative Polymers Used in Construction

There are numerous types of polymers that have successfully been utilized on construction applications including pipes and fittings, foundations, roofing, flooring, paneling, roads and insulation, etc. These polymers often have excellent resistance to environmental elements—they neither rot nor rust, and require very little maintenance. With development of nanotechnology, researchers have been keen on exploring the polymers commonly used in construction industry for their new applications. In the following section, selected nanostructured—Polyurethane (PU), Polystyrene (PS), Polyacrylonitrile (PAN) and Polyvinyl Chloride (PVC) will be discussed. Their nanosynthesis and morphologies are summarized in Table 2.

### 2.1. Synthesis of Nanostructured Polyurethane (NS-PU)

Polyurethane (PU) is a synthetic polymer composed of organic units linked by carbamate bonds. They are traditionally and most commonly formed by reacting a di- or poly-isocyanate with a polyol. Both the isocyanates and polyols used to make polyurethanes contain, on average, two or more functional groups per molecule. There are numerous applications for PUs in everyday life. In construction industry, PUs are mainly used for coatings and building insulation. During the last decades, the application of nanostructured polyurethane (NS-PU) such as polyurethane nanoparticles (PU-NPs) has increased rapidly, as they can be used for various applications, including adhesives and coatings, etc., due to their good adhesion to metallic surfaces, high mechanical performance, and good resistance against chemicals.

PUs are generally synthesized via direct polycondensation of diisocyanate and polyols monomers. In recent years, water-soluble or biocompatible/biodegradable monomers have been more frequently utilized for PU synthesis. Serkis-Rodzen, M. et al., prepared waterborne PU based NPs by polycondensation of polycarbonatediols (PCD), 2,2-bis(hydroxymethyl)propionic acid (DMPA) and 1,6-diisocyanatohexane (HDI) [44]. The formation of PU polymers was carried out in acetone. PU-NPs dispersion were subsequently obtained after removal of acetone. The particle sizes of the prepared PU-NPs were found to be in the range of 60~345 nm and all the prepared PU-NPs could be potentially used as mechanically strong coatings/films.

**Figure 1:**
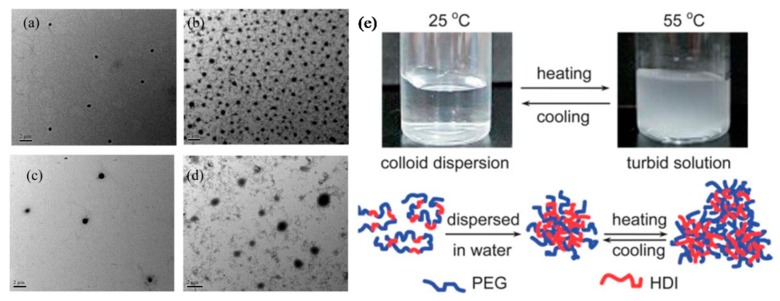
TEM images of LDI-PEG600 at (**a**) room temperature (**b**) 55 °C; HDI-PEG600 at (**c**) room temperature (**d**) 55 °C; (**e**) The optical pictures (upper panel) and possible aggregation process (lower panel) showing transition of HDI-PEG600 upon heating and cooling.

Fu, H. et al. have reported the preparation of temperature-responsive and biodegradable PU-NPs based on l-lysine ethyl ester diisocyanate (LDI) or hexamethylenediisocyanate (HDI) and poly(ethylene glycol) (PEG) [45]. Firstly, PU polymers were synthesized based on a condensation reaction of HDI or LDI with different molecular weight PEG (600, 1500 and 2000) respectively, followed by the preparation of PU-NPs. TEM analysis showed that those PU-NPs were nearly spherical in shape at both room temperature and 55 °C (Figure 1a–d). The as-prepared PU-NPs were found to be temperature-responsive during the heating and cooling cycle process. Moreover, the cloud point temperatures of the PU-NPs could be adjusted by controlling the hydrophilic–hydrophobic balance and the concentration of polymer (Figure 1e).

Wang, A. et al. also used biocompatible PUs to synthesize temperature and pH responsive nanoparticles [46]. Two different methods, dispersion in acid with stirring and dialysis, were used for the fabrication of PU-NPs and the size of the obtained NPs ranges from 250~750 nm. Zanetti-Ramos, B. G. et al. reported the synthesis of PU-NPs by polycondensation with isophorone diisocyanate (IPDI) and natural triol (castor oil), using emulsion technique [47]. The prepared PU-NPs were found to be between 200 and 300 nm in particle size with a polydispersity index ranging from 0.3 to 0.4. Moreover, Shendi, H. K. et al. prepared water-borne PU-NPs by using IPDI and modified sun flower oil with the technique of emulsion polymerization [48]. The film prepared from the waterborne PU-NPs showed excellent thermal and mechanical properties as well as high hardness and tensile strength.

**Figure 2:**
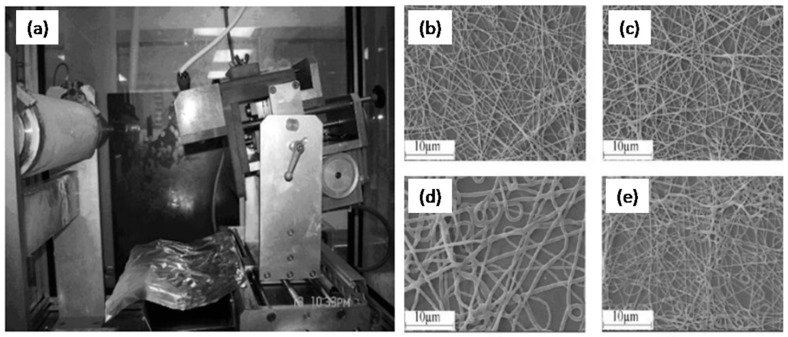
(**a**) Electrospinning setup and SEM images of the nanofibers at different applied voltages: (**b**) 12; (**c**) 15; (**d**) 20; and (**e**) 25 kV.

Besides PU-NPs, polyurethane nanofibers (PU-NFs) are also of great interests to researchers and have been investigated by numerous research groups. In 2008, Zhuo, H. et al. reported the preparation of a PU-NF by electrospinning at ambient temperature [49]. Synthesized poly(*ε*-caprolactone) diol based PU was dissolved in DMF and subsequently electrospun on an aluminum collector with feed rate of 0.04 to 0.10 mm/min (Figure 2a). As shown in the SEM images (Figure 2b–e), it was found that uniform nanofibers without beads could not be obtained until the applied voltage reached 12.0 kV. If the applied voltages were too high, such as 20.0 or 25.0 kV, diameters of the NFs were not uniform, and many loops were formed (Figure 2d,e). Results also showed that the larger diameter NFs were obtained at a higher feeding rate and smaller uniform NFs were observed in lower feeding rate. Moreover, if the concentration of PU solution was above 12 wt %, no jet formation was observed whereas if the concentration was below 3%, only PU droplets formed. The diameters of final prepared PU-NFs were in the range of 50~700 nm with the tuning of parameters.

**Figure 3:**
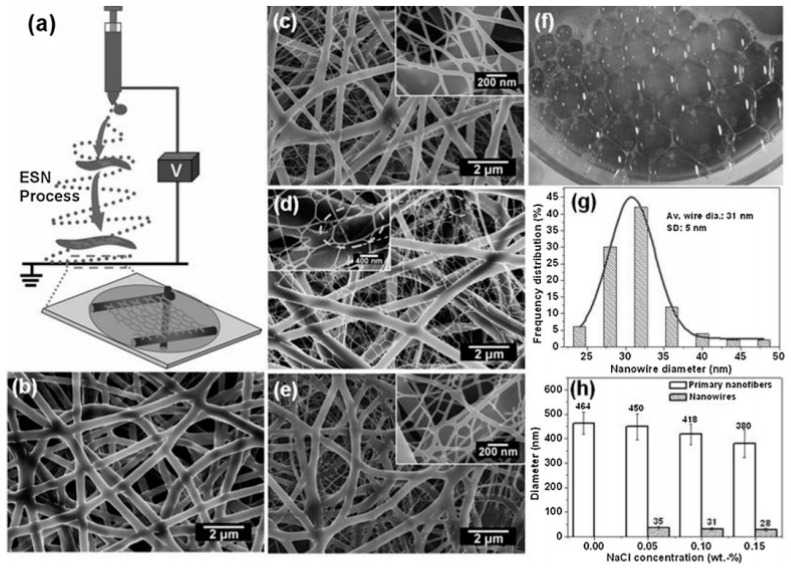
(**a**) Schematic diagrams illustrating the possible mechanism of PU-NF structure formation. FE-SEM images of PU/NaCl PU-NF membranes formed with a voltage of 30 kV and different NaCl concentrations: (**b**) 0 (pristine PU); (**c**) 0.05; (**d**) 0.1 and (**e**) 0.15 wt %; (**f**) Optical image of soap bubbles; (**g**) Histogram showing the nanowire diameter distribution of nano-nets presented in (**d**); (**h**) The effect of NaCl content on the diameter distribution of nanofiber and nanowire.

Hu, J. et al. reported one-step preparation of versatile nanofiber/net membranes by electrospinning technique (Figure 3a) [50]. Besides using a 7 wt % PU DMF solution, PU/NaCl or PU/sodium lauryl sulfonate (SLS) DMF solution were also used in the fabrication. Figure 3b shows that the as-prepared pristine PU fibers have a uniform diameter (464 nm) while the diameter was decreased to 418 nm as NaCl concentration increased. One interesting feature was the presence of soap bubble-like structured nano-nets which had a high coverage rate (over 90%) and layer-by-layer stacked structure (~31 nm). Due to high porosity and large stacking density, such PU-NF based membranes can provide an approach for the construction of ultra-filters, special protective clothing and ultrasensitive sensors, etc.

Lev, J. et al. also reported using electrospinning technique to prepare PU-NFs [51]. The as-prepared nanofibers were collected on polypropylene textiles, as a range of flat sheet non-woven nanofiber membrane layers. The PU-NF base membrane can effectively remove pathogen from waste water upon filtration. Additionally, another work done by Lev, J. et al. also showed the fabrication of 0.25 µm PU-NF based membrane, which was able to remove the bacteria of Escherichia coli effectively in laboratory test [52].

**Figure 4:**
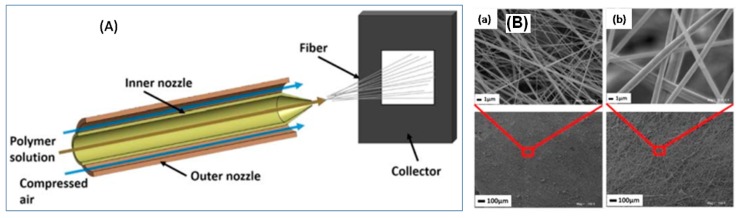
(**A**) Experiment setup of solution blowing and SEM of PU-NFs; (**B**) PU-NF nonwovens with different concentration: (**a**) 10% (**b**) 20%.

Another promising approach to PU-NFs was reported by Guan, K. et al., using solution blowing technique [53]. Typically, the PU DMF solution was delivered to the spinneret and then blown by high pressure airflow, followed by collection. The solution concentration was the key factor of this technique. Low concentration resulted in non-continuous fibers due to the incomplete solvent volatilization, whereas high concentration led to the failure of forming fibers because of poor fluidity. A mass of beads was found for a concentration of 6% whereas beads were hardly found for a concentration of 8%. Once the concentration reached 10%, uniform fibers were found without adhering phenomenon. Similarly, Polat, Y. et al. used solution blowing technique (Figure 4A) to prepare PU-NFs with diameters of 170 ± 112 nm and 671 ± 136 nm using PU DMF solution with a concentration of 10 wt % and 20 wt % (Figure 4B (a,b)), respectively [54].

### 2.2. Synthesis of Nanostructured Polystyrene (NS-PS)

Polystyrene (PS) is a synthetic aromatic polymer made from monomer styrene by single bonds. They are typically formed by undergoing self-polymerization initiated by free radicals and the final PS can be in the state of solid or foam. They are usually used as building insulation materials and various packing materials in construction industry. In recent years, nanostructured PS (NS-PS) has been investigated extensively and applied in many fields due to its promising physical properties and excellent chemical stability.

Generally, PS nanoparticles (PS-NPs) are prepared by direct synthesis using various polymerization techniques. Among these methods, emulsion polymerization is widely used because of its rapid polymerization rate, high conversion, environmentally-friendly and readily scalable characteristics [80]. A typical procedure involves addition of an amount of emulsifier into pure water, followed by styrene monomers. After being stirred for an additional period, initiator is subsequently introduced to the system and the reaction mixture is gradually heated up and maintained at a certain temperature for several hours. The final PS-NPs can be collected by simple filtration. The stirring speed rate, the amount of emulsifier and initiator added, and the reaction temperature can collectively influence the particle size of final PS-NPs.

**Figure 5:**
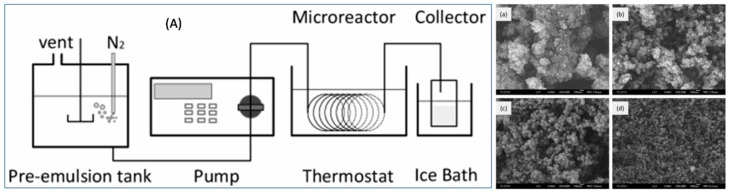
(**A**) Setup for continuous flow emulsion polymerization and SEM images of products obtained. (**a**) Emulsifer TX-100/SDBS, TX-100/SDBS concentration 6.812/8.515 nM, Pre-emulsion 20 min; (**b**) Emulsifier TX-100/SDBS, TX-100/SDBS concentration 6.812/34.06 nM, pre-emulsion 10 min; (**c**) Emulsifier TX-100/SDBS, TX-100/SDBS concentration 6.812/1.703 nM, pre-emulsion 20 min; (**d**) Emulsifier TX-100/SDBS, TX-100/SDBS concentration 27.25/6.812 nM, pre-emulsion 10 min.

Compared to conventional preparation of PS-NPs, Ernawati, L. et al. reported the synthesis of highly dispersed cationic PS-NPs with small diameters [55]. The combined use of 2,2′-azobis[2-(2-imidazolin-2-yl)propane]di-hydrochloride (VA-044) as the initiator and acetone/water system as solvent medium afforded the successful synthesis of cationic PS-NPs. The addition of acetone as a co-solvent was due to its rapid diffusion characteristic and ability to promote the formation of small droplets in emulsion system. The initiator VA-044 was used to control both the surface charge and size of the particles. The final as-prepared cationic PS-NPs have a diameter of 31 nm. Moreover, Liu, X. et al. reported the continuous flow synthesis of PS-NPs using a microflow system [56]. The apparatus setup is shown in Figure 5A. The average size of the PS-NPs obtained is in the range of from 52 to 92 nm and could be facilely adjusted by changing the emulsifier type and concentration (Figure 5a–d). Rapid and reliable flow synthesis of PS-NPs could be achieved by using such a continuous emulsion polymerization in the tubular microreactor.

**Figure 6:**
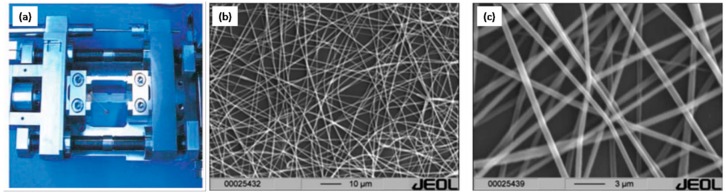
(**a**) Experiment setup of electrospinning technique and SEM images of PS-NFs, (**b**,**c**) in lower and larger magnification.

PS nanofibers (PS-NFs) have been explored as well as one of the important NS-PS materials. Asran, A. Sh. et al. prepared PS-NFs with a diameter range from 150~800 nm (Figure 6b,c) using electrospinning technique [57]. After the successful preparation of the PS-NFs, the investigation of the micromechanical properties and ductile behavior indicated that brittle PS can be modified by using electrospinning synthesis so that a transition from crazing behavior to micronecking and ductile behavior can take place.

Hashemifard, N. et al. made the electrospun PS-NFs as well with 15 *w/v* % PS solution under the voltage of 20 kV [58]. The prepared fibers were applied as adsorbent in solid-phase extraction of disulfine blue from aqueous solutions and it was found that the extraction and preconcentration of disulfine blue in different aqueous samples could be effectively realized. Similarly, Liu, F. et al. reported the electronspun synthesis of PS-NFs with a diameter ranging from 0.25–1 um [59]. Investigation showed that the prepared fibers had large adsorption capacity and were suitable for the application of dispersive liquid–liquid microextraction. Another work of PS-NF based membrane preparation was done by Lee, M. W. et al. by using electrospinning technique as well. The synthesis was carried out from a 20 wt % PS DMF solution under 5 kV volt, leading the formation of NF membranes with an average diameter of 317 nm [60]. Interestingly, the prepared membranes demonstrate superhydrophobicity and superoleophilicity, showing a contact angle of 155° and 0° to water and diesel, respectively.

Li, H. et al. prepared the ultrafine 19 nm-diameter PS-NFs by a facile approach based on the fast freeze technique [61]. A PS dilute solution was initially prepared and was then fast frozen in liquid nitrogen to form white particles. Subsequently, ethanol (at −50 °C) was then added in and the contents maintained at −50 °C for 24 h, resulting in the formation of PS-NF dispersion. PS nanofibrous membranes could be further fabricated by the direct filtration of the dispersion. The PS-NF based membranes had an ultra-high porosity up to 87%, as well as a super-hydrophobic and super-lipophilic surface. Absorption study demonstrated the membranes could absorb methylene blue with very fast and highly efficient rate.

### 2.3. Synthesis of Nanostructured Polyacrylonitrile (NS-PAN)

Polyacrylonitrile (PAN) is a synthetic, semicrystalline organic polymer plastics made from the monomer acrylonitrile. Acrylonitrile is also a co-monomer unit in several important copolymers, such as the known polymers of styrene-acrylonitrile (SAN) and acrylonitrile butadiene styrene (ABS) plastic. PAN is a versatile polymer and is used for various applications in construction industry, i.e., ultra filtration membranes, outdoor awnings and fiber-reinforced concrete, etc. Nanostructured PANs (NS-PANs), especially PAN nanofibers (PAN-NFs) have been widely explored and applied in many fields.

**Figure 7:**
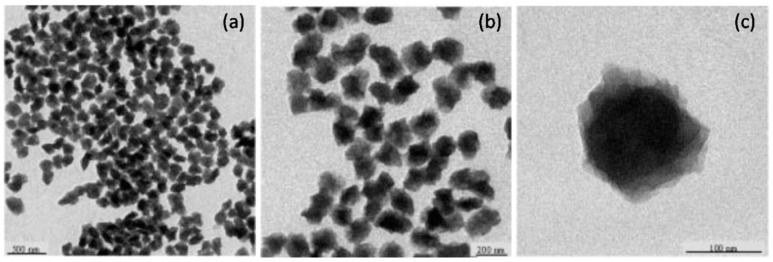
TEM images of PAN-NPs in different scale bar: (**a**) 500 nm (**b**) 200 (nm) (**c**) 100 nm.

The preparation of PAN nanoparticles (PAN-NPs) is usually achieved via different polymerization techniques. Landfester, K. et al. reported the preparation of PAN-NFs via miniemulsion polymerization [62]. As shown in the TEM images (Figure 7), the prepared PAN-NPs had a diameter ranging from 100~180 nm. Jeon, H. J. et al. reported the synthesis of monodisperse spherical PAN-NPs using dispersion polymerization with a poly(*N*-vinyl pyrrolidone) (PVP) complex as both a macroinitiator and a colloidal stabilizer [63]. The average diameters of PAN-NPs synthesized with 20, 30, 40, and 50 wt % of initiator at 30 °C for 24 h were 263.5, 163.1, 157.3, and 143.5 nm, respectively with a slightly crumpled spherical appearance. Zhang, Y. et al. prepared PAN-NPs with the size less than 100 nm using high concentration of acrylonitrile via semi-continuous emulsion polymerization [64]. Their studies revealed that PAN-NPs with smaller sizes (<100 nm) could be fabricated by modestly slowing down the monomer feeding rate, decreasing the monomer concentration, lowering the polymerization temperature, and properly increasing the surfactant amount.

**Figure 8:**
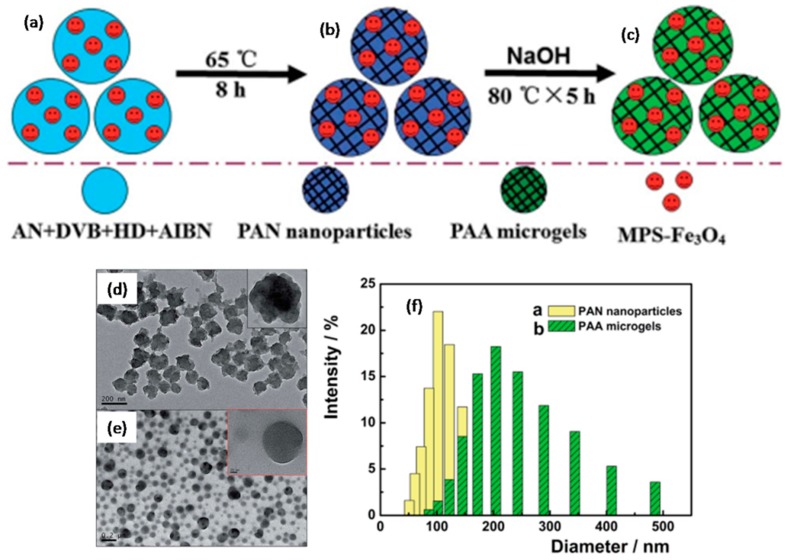
(**a**–**c**) Illustration for the preparation of magnetic PAA nanogels; TEM images of (**d**) PAN-NPs and (**e**) PAA nanogels nanoparticles prepared with 1.8 g DVB; (**f**) Size distributions of PAN-NPs and PAA nanogels.

Lee, I. et al. reported the preparation of PAN-NPs via ultrasonic irradiation assisted microemulsion polymerization and the subsequent modification of the particles with amidine/Schiff base [65]. The resulting modified PAN-NPs could be used for the sensitive and selective detection of free copper ions. Zhang, J. et al. prepared cross-linked PAN-NPs via miniemulsion polymerization [66]. The corresponding poly(acrylic acid) (PAA) nanogels could then be obtained under basic conditions. Moreover, magnetic PAA microgels could be obtained if magnetic nanoparticles (modified Fe_3_O_4_ nanoparticles) were added in oil phase during the synthesis (Figure 8a–c). The typical TEM images of PAN-NPs and corresponding PAA nanogels (Figure 8d,e) showed that PAN-NPs had a uniform size of about 100 nm before hydrolysis, while PAA nanogels shrunk a little with/though the size of nanogels remained below 100 nm. However, it is evident from Figure 8f that both the particle size and size distribution of PAA nanogels clearly increased after hydrolysis.

**Figure 9:**
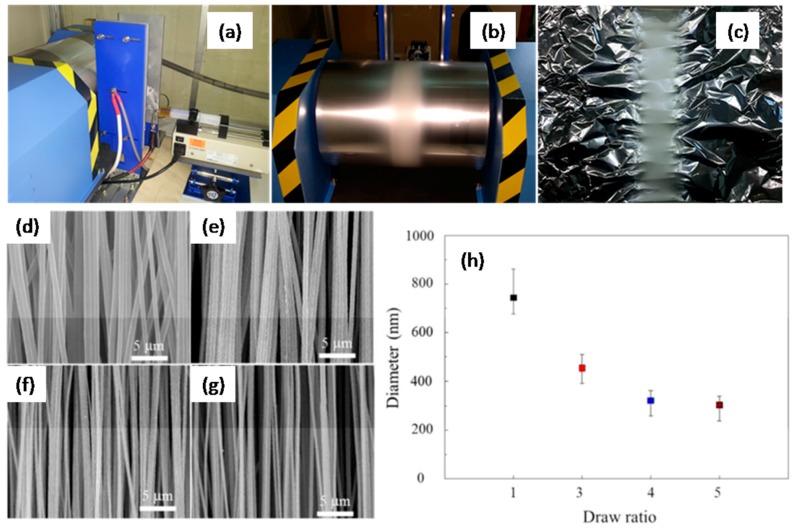
Electrospinning setup (**a**), and collected PAN nanofibers on the rotating drum with alignment to one direction (**b**,**c**). SEM images of aligned and drawn PAN-NFs with different draw ratio (**d**) 1 (as-electrospun), (**e**) 3, (**f**) 4, and (**g**) 5. (**h**) Variation of fiber diameter as a function of stretch ratio.

PAN nanofibers (PAN-NFs) belong to another important class of NS-PAN materials and their preparation and modification have been widely investigated. Youm, J. S. et al. prepared PAN-NFs by typical electrospinning synthesis [67]. In order to improve uniaxial orientation of the polymer chains, the electrospun PAN-NFs were drawn in one direction along the fiber axis (Figure 9a–c). The drawing was conducted in two steps. Samples were first drawn uniaxially in water in the temperature range of 90–95 °C, and they were then further drawn uniaxially under dry hot air at a temperature of 160 °C. Figure 9d–h shows the SEM images and diameter variation graph of PAN-NFs before and after stretching. The results revealed that the two-step drawing process could improve the crystalline properties and the molecular chain orientation of the PAN-NFs, hence enhancing their mechanical properties. Kim, H. Y. et al. also prepared PAN-NFs as using general electrospinning technique [68].

Wang, W. et al. reported an efficient and convenient method for the synthesis of modified PAN-NFs with antibacterial property [69]. PAN polymer was first prepared via radical polymerization. PAN, NaN_3_, AgNO_3_ were then mixed in DMF solvent and stirred at 120 °C for 4.5 h. The resulting solution was processed by electrospinning technique to make modified PAN-NFs with a diameter of 50–100 nm. Silver ion was incorporated into PAN matrix via Click chemistry, resulting in the dramatic enhancement of antibacterial property of the prepared PAN-NFs. Almasian, A. et al. also made surface modification of electrospun PAN-NFs using amine and it was found that the modified PAN-NFs were promising candidates for anionic dye adsorption from colored wastewater with high adsorption capacity [70]. Similarly, Dilparzir, S. et al. prepared TiO_2_ doped PAN-NF based membrane using electrospinning synthesis, followed by further treatment with UV-ozone [71]. Electrospun PAN-NFs prepared by Makaremi, M. et al. were functionalized with zinc oxide (ZnO) nanoparticles and coated with a layer of chitosan [72]. The modified PAN-NFs exhibited greatly improved mechanical properties and excellent anti-bacterial and water filtration performance. Moreover, Elkhaldi, E. A. et al. reported the preparation of PVDF nanoparticle incorporated PAN-NFs by submersing the electrospun PAN-NFs in PVDF solution and then by post-heating treatment [73]. It was found the mechanical properties of the PAN-NF membranes were remarkably improved without great trading off the membrane’s flux performance.

**Figure 10:**
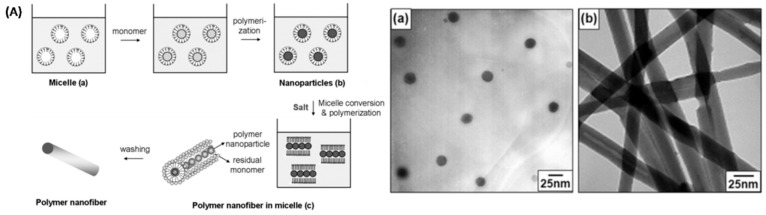
(**A**) Synthetic procedure of PAN-NFs using microemulsion polymerization TEM images of (**a**) PAN-NPs with an average diameter of 20 nm and (**b**) PAN-NFs with an average diameter of 25 nm with 0.3 M DoTAB.

In addition to widely employed electrospinning technique, other approaches to preparation of PAN-NFs have also been explored by researchers. Jang, J. et al. reported the facile preparation of PAN-NFs using microemulsion polymerization [74], and the overall procedure of PAN-NF preparation is given in Figure 10A. TEM images in Figure 10a,b revealed that the diameters of the PAN-NPs and PAN-NFs formed were 20 and 25 nm, respectively. It was believed that the introduction of ferric chloride to the system was crucial, generating coordination between the CN groups and iron as a prerequisite for PAN-NF formation.

### 2.4. Synthesis of Nanostructured Polyvinyl Chloride (NS-PVC)

Polyvinyl chloride (PVC) is one of the world’s most widely produced synthetic plastics which are made from monomer vinyl chloride by single bonds linkage. Pure PVC is a white, brittle solid and exists commonly in two basic forms: rigid and flexible. The rigid PVC is usually used for pipe and floor coatings in construction industry. The flexible PVC can used for plumbing, electrical cable insulation, inflatable products, etc. Similarly, nanostructured PVCs (NS-PVCs) have been investigated, reported and employed in applications.

Vatani, Z. et al. reported the fabrication of 40 nm PVC-NP with polyaniline at the presentence of various surfactant to form nanocomposites [75]. The process demonstrated a novel approach to modification of PVC-NPs.

PVC nanofibers (PVC-NFs) have also been prepared and studied. Phatcharasit, K. et al. reported the preparation of PVC-NFs and its corresponding nanofaborous membrane [76]. The synthesis of PVC-NFs was done by typical electrospinning with a diameter of 100 nm. Jiang, T. et al. reported the preparation of electrospun CPVC-NF nonwovens as well [77]. Their findings showed a diameter of 135 nm of CPVC-NFs could be achieved. The resulting CPVC-NF nonwovens also demonstrated good performance of surface potential storage and high filtration efficiency. Krupa, A. et al. studied the surface properties of electrospun PVC-NFs which was modified by the treatment of plasma [78]. Their results revealed that when the PVC-NF based mat was treated by plasma for 60 s, the mat lost its hydrophobic properties as evidenced by the contact angle change from 130° to <2°.

**Figure 11:**
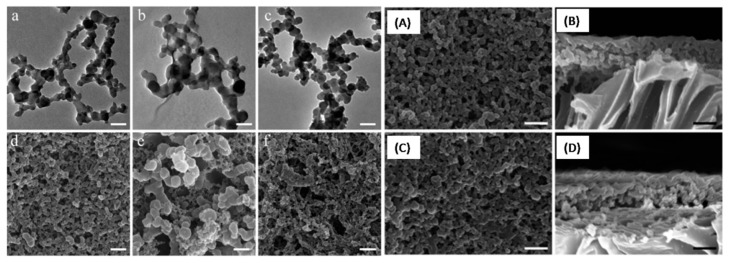
TEM and SEM images of the PVC-NFs made in (**a**,**d**) DMAC, (**b**,**e**) NMP, and (**c**,**f**) DEC solvent and SEM images of PVC mesoporous membranes: top and cross-setion view of the membrane made from (**A**,**B**) 12 mL and (**C**,**D**) 14 mL of the nanofiber dispersion, respectively.

Similar to PS-NFs discussed previously, Guo, N. et al. reported the preparation of novel PVC-NF based mesoporous membranes prepared via a modified freeze-extraction technique [79]. Typically, a homogeneous PVC solution was prepared in a solvent (DEC, DMAC, or NMP) at 40 °C, followed by rapidly frozen in liquid nitrogen. Then ethanol was added at −40 °C and the system were maintained at −40 °C for 24 h to form the PVC-NF dispersion. The resulting mesoporous PVC membranes could be fabricated by a direct filtration of the PVC-NF dispersion. SEM and TEM (Figure 11a–f) showed that the prepared PVC-NFs made in *N*,*N*-dimethylacetamide (DMAC) were uniform and had an average diameter of ~45 nm. Additionally, the SEM images (Figure 11A–D) of the prepared PVC membranes displayed a thickness, ranging from 360 nm to 1055 nm, and a high porosity of up to 63%, which is at least 5 times greater than that of most of commercial ultrafiltration membranes. Moreover, the membrane also had high hydrophobicity and superoleophilicity, essential for oily waste water treatment.

## 3. Building Applications of Polymers Used in Construction Industry

Polymers that are widely used in construction and their nanostructured materials have been briefly introduced in the previous section. Their nanosynthesis and nanostructures have been described as well. In the following section, building applications of these nanostructured polymers will be summarized (Table 3).

### 3.1. Air Filtration

**Figure 12:**
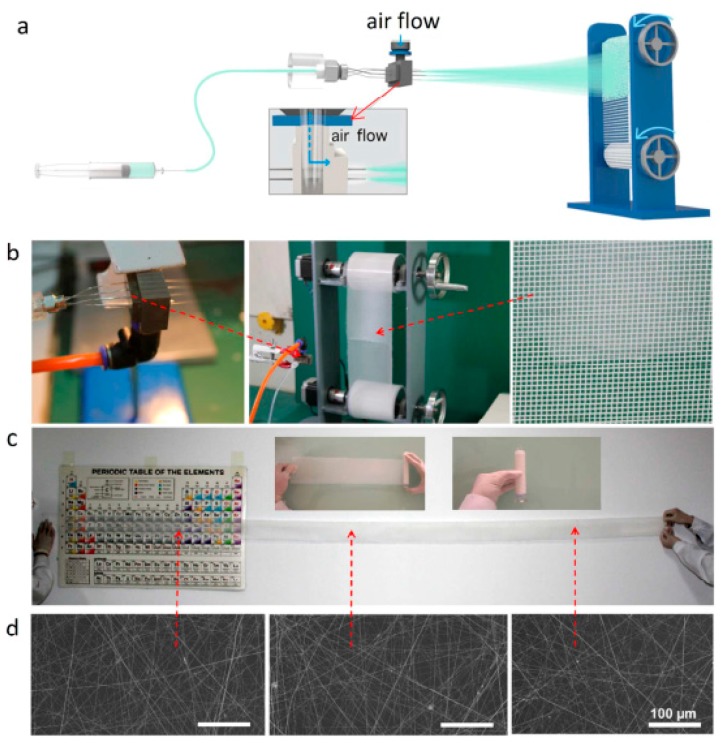
Roll-to-roll production of nanofibers. (**a**,**b**) Experimental setup; (**c**) A transparency check of deposited fibers; (**d**) SEM images of the blowspuns taken from three different places of the coated rolling mesh.

**Figure 13:**
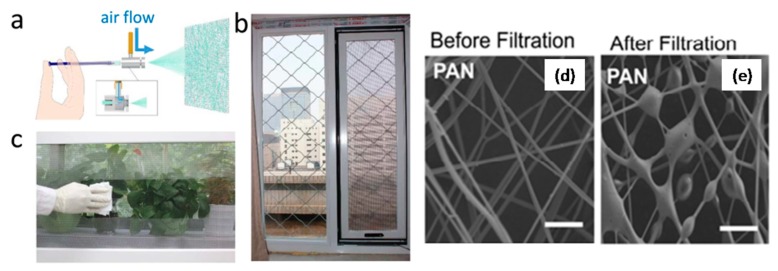
Real window based PM filtering performances. (**a**) Practical model of the blowspinning setup for window screen coating; (**b**) Real window coated with PAN-NFs; (**c**) Successful wiping of nanofibers from the window screen with a tissue paper and SEM images of PAN-NFs (**d**) before and (**e**) after filtration.

Particle pollution is a mixture of solids and liquid droplets floating in the air and it has caused many serious public health issues. Therefore, air filtration, such as particulate matter (PM 2.5) removal, has been of great importance to the living quality and health of people. Wu, H. et al. developed a fast, efficient and free-of-high-voltages technique via blow-spinning for the large-scale direct coating of PAN-NFs onto window screen for indoor PM pollution protection [81]. Typically, a continuous blowing of 10 wt % PAN DMF solution onto a commercial Nylon mesh, rolling with a certain speed resulted in uniformly arranged transparent fiber’s coating (Figure 12a–d). The SEM images of the prepared PAN-NFs were examined before and after PM 2.5 removal (Figure 13d,e). Results showed that the fibers had a diameter of 150–250 nm before filtration. When PMs strongly adhere to the surface of PAN-NFs, an initial layer coating was formed and subsequently turned into stable spherical shape at the junctions of the fibers. This results in an increase in the fiber’s diameter from 150–200 nm to several hundred nm. Finally, real windows based filtering performance was examined (as shown in Figure 13a–c) and it was found that by installing the blowspun PAN-NFs on real window screen, minimum 90.6% removal efficiency of PM 2.5 was achieved in an extremely hazardous weather over 12 h.

Jing, L. et al. reported the preparation of ionic liquid modified PAN-NFs by electrospinning [82]. Highly viscous and hydrophobic ionic liquid diethylammonium dihydrogen phosphate (DEAP) was involved in the fabrication. The most important was that the modified PAN-NFs demonstrated a superior PM 2.5 capture capacity due to the surface roughness and improved hydrophilicity induced by the addition of DEAP. Zhang, R. et al. developed high-efficiency (>99.5%) polyimide-nanofibers (PI-NFs) for the high temperature PM 2.5 removal [83]. The electrospun PI-NFs showed a diameter of 300 nm and exhibited high thermal stability. Surprisingly, the PM 2.5 removal efficiency of the prepared PI-NFs was kept unchanged in the temperature range of 25–370 °C. Further investigation showed that these fiber based membranes had high air flux with very low pressure drop and could continuously work for >120 h for PM 2.5 index >300. A field test also showed that the PI-NFs could effectively remove >99.5% PM particles from car exhaust at high temperature. Additionally, Xu, J. et al. used roll to roll technique to transfer electrospun Nylon 6 nanofibers (N6-NFs) from roughed metal foil to a receiving mesh substrate, hence successfully preparing a transparent air filter system [84]. Compared to conventional electrospinning, transfer method was 10 times faster in making a nanofiber film which had a better filtration performance owing to a better uniformity. The as-prepared transparent air filter demonstrated a result of >99.97% removal of PM 2.5 at ~73% of transmittance. Similarly, Li, Q. developed polycarbonate nanofiber (PC-NF) based membrane for high efficiency particulate matter filtration [85]. Under the experiment conditions, PC-NF membrane gained ~100% filtration efficiency due to its high specific surface area and polarity.

In addition, PNF based nanocomposites have also been reported in the development of air filtration systems. Zhang, S. et al. reported the preparation of polyacrylonitrile/polysulfone (PAN/PSU) composite based membranes for air filtration [86]. The prepared membranes inherit small pore size and controllable packing density. A high filtration efficiency of 99.992% could be achieved under the experiment conditions. Wang, S. et al. reported a novel nanofibrous membranes based on polyvinylidene fluoride (PVDF) nanofibers incorporated with polytetrafluoroethylene nanoparticles (PTFE NPs) which could exhibited a low pressure drop and a high filtration efficiency as high as 99.972% [87]. Another interesting work done by Su, J. et al. was the fabrication of TiO_2_ doped PAN-NFs for air filtration. The prepared nanofiber membrane was shown to exhibit high filtration efficiency and high photocatalytic activities at the same time [88].

### 3.2. Thermal Energy Storage

Storing heat from renewable thermal energy sources for consumption at a later time and a different location has been of great interests on energy storage. Phase change materials (PCMs) can effectively store thermal energy during phase changes and have been used in buildings to enhance the thermal comfort of lightweight buildings for energy savings. Therefore, the storing and releasing of latent heat energy through the melting and solidifying of phase change materials (PCMs) has become the most efficient and cost-effective way for a variety of thermal energy storage applications. PS-NPs, especially PS encapsulated PCM nanocapsules, have been developed rapidly for the purpose of thermal energy storage. Sari, A. et al. reported the preparation of n-tetracosane and *n*-octadecane mixture contained PS micro/nanoparticles for low-temperature latent heat thermal energy storage applications [89]. The PCM encapsulated PS capsules were synthesized by emulsion polymerization with a particle size range of 10 nm to 115 um. It was found the resultant capsules had a melting temperature of 25.96 °C and latent heat of 156.39 J·g^−1^, showing good performance for latent heat thermal energy storage at low temperature. Similarly, Fang, Y. et al. also prepared octane contained PS-NPs via ultrasonic-assisted microemulsion polymerization [90]. The diameter of the nanoparticles was in the range of 100 nm to 123 nm. Thermal characterization suggested that latent heat of the PS-NPs was 115 kJ/kg. Cho, W. et al. made PCM PS-NPs via miniemulsion and the resultant nanoparticles showed a diameter of ~250 nm and good thermal storage properties [91].

**Figure 14:**
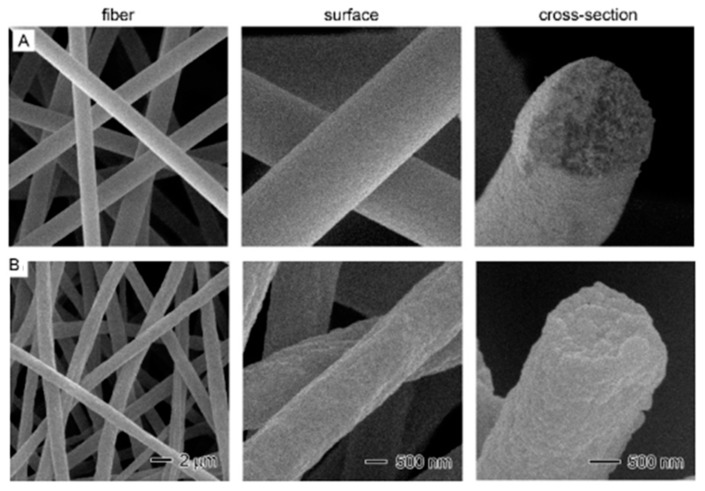
SEM images showing the overview (left column), surface (middle) and cross-section (right) of PS-NFs with different LA/PS ratios: (**A**) pure PS; (**B**) LA0.25PS.

In addition to the widely used PNPs, PNFs have also been used in thermal energy storage materials. Lu, P. et al. reported a simple and reliable approach to the fabrication of lauric acid (LA)-containing PS-NFs [92]. The fiber composites were fabricated by co-electrospinning synthesis. As examined by SEM (Figure 14A,B), the neat PS-NF yarns an average diameter with a broad size distribution range (1.93 ± 0.24 µm) among all the samples whereas the LA4PS (LA to PS ratio 4:1) had an average diameter of 1.3 um. Thermal energy storage performance of LAPS nanofibers was measured at T4P mode instead of the traditional T1 mode. It was noticeable that the PS-NF composite could achieve a 78.4% storage capacity, which was higher than the previous reported values (<50%). Similarly, Mu, S. et al. used poly(styrene-*co*-acrylonitrile) (PSAN) and LA PCM to make nanofibers via electrospinning [93]. Their investigation showed that NFs had moderate temperature, enthalpy of phase transition and good ability to store thermal energy. Ke, H. reported the preparation of methyl stearate (MES)—polyethylene terephthalate (PET) nanofiber (PET-NF) composite by eletrospinning synthesis for thermal energy storage [94]. It was found that the weight percentage of MES could be as high as 50% without leakage in its liquid state. The maximum melting enthalpy of the prepared nanofiber composite could reach up to about 90.43 kJ/kg, indicating good storage capacity of thermal energy. Kook, J. W. et al. developed octane@PU nanocapsules incorporated poly(ethylene oxide) nanofibers (PEO-NFs) [95]. Thermal property test showed that the latent heat capacity of the resultant electrospun PEO-NFs increased with the increase in content of nanocapsules.

### 3.3. Sound Absorption

**Figure 15:**
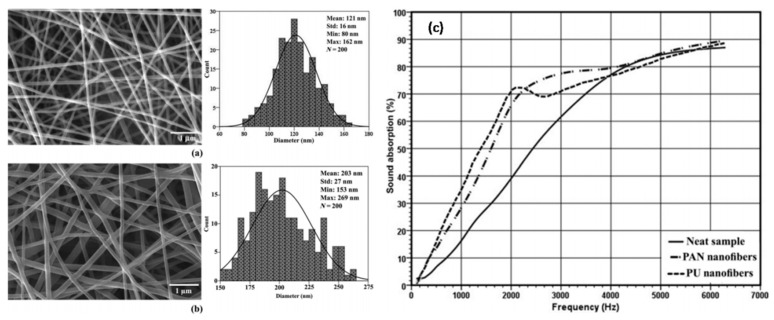
SEM images (left) and diameter distribution (right) of (**a**) PAN-NFs and (**b**) PU-NFs. (**c**) Sound absorption curves of neat sample (without nanofibers), nonwoven with PAN-NFs and nonwoven with PU-NFs.

It is well known that noise commonly have serious adverse effects on both human beings and machines, so noise is often referred to as a form of pollution and is the object of environmental regulations. Rabbi, A. et al. reported the use of PAN-NFs or PU-NFs incorporated in PET nonwovens as highly effective sound absorbers [96]. First, the PAN-NFs and PU-NFs were fabricated using electrospinning synthesis then collected and dried in an oven. Morphology of the prepared fibers were characterized by SEM as shown in Figure 15a,b. The average diameters of PAN and PU nanofibers are 121 ± 16 nm and 203 ± 27 nm, respectively. It was found that the addition of nanofiber layers could effectively lead to an increase in the sound absorption coefficient at low frequencies whereas incident sound waves could still pass through them (Figure 15c). Because of the easier electrospinning, and better solubility of PAN polymer comparing with PU polymer, PAN-NFs is preferred from industrial point of view. Similarly, Bahrambeygi, H. et al. prepared PU-NFs and PAN-NFs and incorporated them into PU foam for sound absorption [97]. The results showed that the presence of nanofibers within PU foam led to considerable enhancement of sound absorption coefficient at all ranges of frequencies, especially at low frequencies. Xiang, H. et al. prepared electrospun PAN-NFs and its corresponding porous membranes [98]. It was found that the anti-noise property of the resultant PAN-NFs incorporated traditional acoustical materials (perforated panel, foam and fiber) could be greatly enhanced especially in the low and medium frequency range. Another interesting work was done by Wu, C. M. et al., and PVDF-NFs and PVDF-NFs/CNT were used to fabricate corresponding membrane using electrospinning [99], showing that the PVDF-NF membrane could absorb sound waves in a low-frequency region. In contrast, sound absorption of the PVDF/CNT could be achieved at low-frequency region, indicating that electrospun PVDF-NFs covered acoustic foam was an efficient sound absorber because of its favorable absorption performance.

### 3.4. Other Applications

In addition to the application discussed above, there are other building applications achieved by NSPs as well, some of them are summarized below:

#### 3.4.1. Anti-corrosion Coating

**Figure 16:**
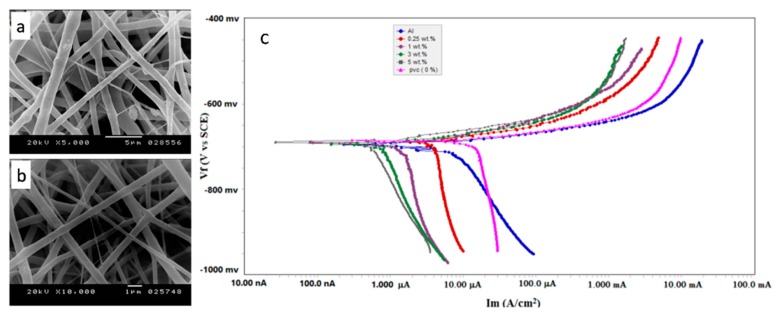
SEM images of (**a**) neat PVC-NFs (**b**) creia embedded PVC-NFs and (**c**) Tafel curves for electrochemical experiments of aluminium coated with PVC-NFs with and without addition of ceria in 0.1 M HCl.

Corrosion is a natural process. The gradual destruction of materials (usually metals) by chemical and/or electrochemical reaction with their environment, causes huge economic losses. Gaballah, S. et al. developed new, cost effective and high performance creia PVC-NF nanocomposites for corrosion resistance of aluminium [100]. The PVC-NFs were prepared by electrospinning synthesis and collected on the metal collector subsequently. The prepared neat PVC-NFs and creia PVC-NF composites had a diameter of 620 nm and 428 nm, respectively (Figure 16a,b). Cyclic potentiodynamic polarization measurements show that coating the Al surface with PVC-NFs could reduce both corrosion currents, and corrosion rates, and increase the polarization resistances compared to uncoated surface, indicating the prepared PVC-NF composite coating could protect to a great extent of aluminum against corrosion in 0.1 M HCl solution (Figure 16c).

#### 3.4.2. Metal Coating

Due to their good adhesion to metallic surfaces and excellent mechanical properties, PU-NPs have been developed as metal adhesives or coatings. Müller, K. et al. prepared PU-NPs with high molecular weight via nonaqueous emulsion polyaddition [101]. The average particle sizes of PU-NPs were found to be in a range of 35 to 95 nm. These PU-NPs were then fabricated for thin coatings on metallic surfaces. Results revealed that the usage of PU-NPs was significantly reduced to only 25% of traditional material that is used for same applications.

#### 3.4.3. Reinforced Adhesive

Razavi, S. M. J. et al. prepared smooth, continuous PAN-NFs with a diameter of 362 ± 87 nm using electrospinning [102]. The fibers were subsequently incorporated in an epoxy-based adhesive layer to improve the adhesive joint’s mechanical performance. The determination of fracture energy of the adhesive demonstrated that an improvements of 127% in fracture energy could be achieved, indicating an outstanding enhancement for the mechanical property of the epoxy adhesive.

#### 3.4.4. Flame Retardant

In construction industry, flame retardants are a key component in reducing the devastating impact of fires on people, property and the environment. They are commonly added to potentially flammable materials, including textiles and plastics. Xiao, L. et al. reported the preparation polyamide 66 nanofibers (PA66-NFs) incorporated with nanoscale grapheme hybridized with red phosphorus (NG-RP) [103]. The novel electrospun fiber composites have a core-shell structure with a diameter ranging from 225–425 nm. The mechanical properties and flame-retardancy of the PA66-NF composite were considerably improved.

## 4. Outlook and Conclusions

Polymers are essential materials in construction industry given their many inherent structural and mechanical properties. The incorporation of nanotechnology into polymeric materials allows for greater improvement in these vital properties. In this review, nanostructured polymers, especially polymer nanoparticles and polymer nanofibers have been introduced for their general fabrication techniques. In addition, nanosynthesis and morphologies of widely used polymers in construction, including PU, PS, PAN and PVC, have been highlighted for their nanostructured materials. Polymer nanoparticles are generally prepared by direct synthesis via various polymerization techniques whereas polymer nanofibers are mostly fabricated by electrospinning synthesis. Finally, building applications of a wide variety of nanostructured polymers, such as air filtration, thermal energy storage, sound absorption as well as some of other applications are reviewed. It can be predicted that the field of nanostructured polymers in construction industry will be gradually transformed from a budding stage to a blossoming stage and more fundamental research is required to develop new polymeric nanomaterials in a large scale. These efforts will eventually speed up the commercial utilization of nanostructured polymers in construction.

## Figures and Tables

**Table 1:** Polymers Commonly Used for Building Applications.

Polymer	Applications in Buildings
Polystyrene (PS)	Roof insulation and masonry wall insulation
Polyurethane (PU)	Wall and roof insulation, curtain wall panels, insulation of industrial pipes and storage tanks, sandwich panels.
Polyvinyl chloride (PVC)	Sandwich structured panel and foam layer in coated fabric flooring
Polyacrylonitrile (PAN)	Gas filtration membranes, outdoor awnings, fiber reinforced concrete
Low-density poly ethylene (LDPE)	Film (packaging, agricultural film), extrusion coating (wires and cables), utensils
High-density poly ethylene (HDPE)	Liquid storage (tanks, drums), containers, pipes and extruded profiles, hospital material
Polyester (PET)	Sandwich panel and polyester coated concrete
Polypropylene (PP)	Containers, electrical appliance frames, tubes and geo-membranes
Acrylonitrile butadiene styrene (ABS)	Tubing and conduits
Polyvinyl acetate (PVAc)	Thermoplastic adhesives
Polyvinylidene fluoride (PVDF)	Electrical wire insulation
Polyimide (PI)	Electrical wire insulation
Polyamide (PA)	High performance fibers

**Table 2:** Nanostructured PU, PS, PAN and PVC.

Polymers	Synthesis	Morphology	Size (nm)	Notes	Ref.
NS-PU	Condensation	Particle dispersion	60–345	Acetone (s), 60 °C (T), 4 h (t)	[44]
	Nanoprecipitation; Sonication	Particle	280–300 & 330–450	Nanoprecipitation: Acetone (s), water (s), r.t. (T), 24 h (t)	[45]
				Sonication: water (s), r.t. (T), 2 min (t)	
	Stirring in acid; Dialysis	Particle	250–750	Stirring in acid: HCl (s), r.t. (T)	[46]
				Dialysis: DMF (s), Water (s), r.t. (T)	
	Miniemulsion	Particle	200–300	Water (s), 60 °C (T), 4 h (t)	[47]
	Emulsion	Particle	80–130	Water (s), 80 °C (T), 2 h (t)	[48]
	Electrospinning	Fiber	50–700	DMF (s), 3–12 wt % (c), 12–25 kV (V), 0.04–0.1 mm/min (f.r.), 15 cm (d)	[49]
	Electrospinning	Fiber	418–464	DMF (s), 7 wt % (c), 40 kV (V), 3 mL/h (f.r.), 15 cm and 25 cm (d)	[50]
	Electrospinning	Fiber	80–250	DMF (s), 75 kV (V), 21 cm (d)	[51]
	Electrospinning	Fiber	250	DMF (s), 13.5 wt % (c), 75 kV (V), 21 cm (d)	[52]
	Solution blowing	Fiber	100–400	DMF (s), 6–12 wt % (c), 0.15–0.3 MPa gas pressure, 50 °C (T)	[53]
	Solution blowing	Fiber	170 ± 112 & 671 ± 136	DMF (s), 10, 15, 20 wt % (c), 1–6 bar gas pressure, 15–50 cm (d)	[54]
NS-PS	Emulsion	Particle	~31	Acetone/water (s), 60 °C (T), 2–14 h (t)	[55]
	Emulsion	Particle	52–92	Water (s), 90–95 °C (T), 7–20 min (t)	[56]
	Electrospinning	Fiber	150–800	DMF/THF (s), 20 wt % (c), 20 kV (V), 1 mL/h (f.r.), 15 cm (d)	[57]
	Electrospinning	Fiber	380–500	DMF/THF (s), 15 wt % (c), 20 kV (V), 0.1 mL/h (f.r.), 15 cm (d)	[58]
	Electrospinning	Fiber	250–1000	DMF (s), 15 wt % (c), 20 kV (V), 0.8 mL/h (f.r.)	[59]
	Electrospinning	Fiber	317	DMF (s), 20 wt % (c), 20 kV (V), 0.18 mL/h (f.r.)	[60]
	Fast freeze	Fiber	19	DCE (s) or CH (s), 0.01 wt %, frozen in liq. N_2_, −50 °C (T)	[61]
NS-PAN	Miniemulsion polymerization	Particle	100–180	Water (s), 55 °C (T), 4 h (t)	[62]
	Dispersion polymerization	Particle	143.5–263.5	Water (s), 30 °C (T), 24 h (t)	[63]
	Emulsion polymerization	Particle	<100	Water (s), 67 °C (T), 5 h (t)	[64]
	Microemulsion polymerization	Particle	40–50	Water (s), r.t. (T), 10 min (t)	[65]
	Miniemulsion polymerization	Particle	105–230	Water (s), 65°C (T), 8 h (t)	[66]
	Electrospinning	Fiber	302–744	DMSO (s), 12 wt % (c), 17–23 kV (V), 1 mL/h (f.r.)	[67]
	Electrospinning	Fiber	575	DMF (s), 12 wt % (c), 18 kV (V), 1 mL/h (f.r.)	[68]
	Electrospinning	Fiber	50–100	DMF (s), 6.25 wt % (c), 20 kV (V), 1.25 mL/h (f.r.), 15 cm (d)	[69]
	Electrospinning	Fiber	230–330	DMF (s), 10 wt % (c), 17 kV (V), 1.2 mL/h (f.r.), 16 cm (d)	[70]
	Electrospinning	Fiber	225–335	DMF (s), 9 wt % (c), 15 kV (V), 1.2 mL/h (f.r.), 15 cm (d)	[71]
	Electrospinning	Fiber	450–550	DMSO (s), 8 wt % (c), 13–14 kV (V), 1.4 mL/h (f.r.), 15 cm (d)	[72]
	Electrospinning	Fiber	294	DMF (s), 8 wt % (c), 27 kV (V), 4 mL/h (f.r.), 13 cm (d)	[73]
	Microemulsion	Fiber	20–50	Water (s), r.t. (T), 4.5 h (t)	[74]
NS-PVC	Modification	Particle	108–215	H_2_SO_4_ (s), r.t. (T), 5 h (t)	[75]
	Electrospinning	Fiber	100	DMF/THF (s), 10 and 15 wt % (c), 12–15 kV (V), 0.5 mL/h (f.r.), 12–18 cm (d)	[76]
	Electrospinning	Fiber	100–180	DMF/THF (s), 14–20 wt % (c), 20–29 kV (V), 0.2 mL/h (f.r.), 15 cm (d)	[77]
	Electrospinning	Fiber	600–800	DMF/THF (s), 9 wt % (c), 12 kV (V), 1 mL/h (f.r.), 12 cm (d)	[78]
	Freeze-extraction	Fiber	45	DCE (s), DMAC (s) or NMP (s), 0.01 wt % (c), frozen in liq. N_2_, −50 °C (T)	[79]

c—concentration; d—distance; f.r.—feed rate; s—solvent; T—temperature; t—time; V—voltage.

**Table 3:** Representative Building Applications of Polymers Used in Construction Industry.

Polymers	Building Application	Synthesis	Morph.	Size (nm)	Notes	Performance	Ref.
PAN	Air filtration	Blow spinning	Fiber	150–250	DMF (s), 10 wt % (c)	Minimum 90.6% removal efficiency of PM 2.5 over 12 h.	[81]
PAN	Air filtration	Electrospinning	Fiber	200	DMF (s), 6 wt % (c), 18 kV (V), 4 mL/h (f.r.), 20 cm (d)	Significantly improved PM 2.5 capture capability	[82]
PI	Air filtration	Electrospinning	Fiber	300	DMF (s), 15 wt % (c)	High temperature PM 2.5 removal with high efficiency (>99.5%)	[83]
Nylon-6	Air filtration	Electrospinning	Fiber	100	Formic acid (s), 20% (c), 15 kV (V), 0.06 mL/h (f.r.), 15 cm (d)	>99.97% removal of PM 2.5 at ~73% of transmittance	[84]
PC	Air filtration	Electrospinning	Fiber	319 ± 27	Chloroform (s), THF/DMF (s), 14% and 16% (c), 18 kV (V), 0.06 mL/h (f.r.), 20 cm (d)	high filtration efficiency of 100%	[85]
PAN/PSU	Air filtration	Electrospinning	Fiber	300–500	DMF (s), 9 wt % for PAN(c), 22 wt % for PSU, 30 kV (V), 1 mL/h (f.r.), 20 cm (d)	high filtration efficiency of 99.992%	[86]
PVDF	Air filtration	Electrospinning	Fiber	510	DMF (s), 22 wt % (c), 30 kV (V), 0.5mL/h (f.r.), 15 cm (d)	high filtration efficiency of 99.972%	[87]
PAN	Air filtration	Electrospinning	Fiber	900	DMF (s), 12 wt % (c), 12 kV (V), 60mm/min (f.r.), 12 cm (d)	high filtration efficiency of 97%	[88]
PS	Thermal energy storage	Emulsion polymerizaiton	Particle	10–115 × 10^3^	Water (s), 70 °C (T), 6 h (t)	Good for latent heat energy storage at low temperature	[89]
PS	Thermal energy storage	Microemulsion polymerizaiton	Particle	100–123	Water (s), 65 °C (T), 5 h (t)	Good for latent heat energy storage	[90]
PS	Thermal energy storage	Miniemulsion polymerizaiton	Particle	250	Water (s), 80 °C (T), 24 h (t)	Good for thermal energy storage	[91]
PS	Thermal energy storage	Electrospinning	Fiber	1300–1930	DMF (s), 20 wt % (c), 15 kV (V), 1 mL/h (f.r.), 25 cm (d)	78.4% energy storage capacity	[92]
PSAN	Thermal energy storage	Electrospinning	Fiber	-	DMF (s), 20 wt % (c), 11–17 kV (V), 0.04 mm/s (f.r.), 20–35 cm (d)	Good ability to store thermal energy	[93]
PET	Thermal energy storage	Electrospinning	Fiber	282–500	DMF (s), 12 wt % (c), 16 kV (V), 2 mL/h (f.r.)	Good storage capacity of thermal energy	[94]
PEO	Thermal energy storage	Electrospinning	Fiber	200	Water (s), 5 wt % (c), 10–11 kV (V), 0.18 mL/h (f.r.), 10 cm (d)	Thermal energy storage capacity	[95]
PU & PAN	Sound absorption	Electrospinning	Fiber	121 ± 16 & 203 ± 27	For NS-PAN: DMF/THF (s), 10 wt % (c), 12 kV (V), for PU:NS-PAN: DMF/THF (s), 9 wt % (c), 16 kV (V), 0.25 mL/h (f.r.); 12 cm (d)	Sound absorption coefficient at low frequencies; NS-PAN preferred	[96]
PU & PAN	Sound absorption	Electrospinning	Fiber	-	For NS-PAN: DMF/THF (s), 10 wt % (c), 12 kV (V), for PU:NS-PAN: DMF/THF (s), 9 wt % (c), 16 kV (V), 0.25 mL/h (f.r.); 12 cm (d)	Enhancement of sound absorption coefficient at all ranges of frequencies	[97]
PAN	Sound absorption	Electrospinning	Fiber	333 ± 58	DMF (s), 8 wt % (c), 10 kV (V), 1.2 mL/h (f.r.), 13 cm (d)	Enhancement of sound absorption in low and medium frequency range	[98]
PVDF	Sound absorption	Electrospinning	Fiber	138–156	DMF (s), 18 wt % (c), 20 kV (V), 0.5 mL/h (f.r.), 20 cm (d)	Efficient for sound absorption	[99]
PVC	Corrosion Inhibition	Electrospinning	Fiber	486 & 620	THF (s), 12 wt % (c), 20 kV (V), 9 mL/h (f.r.), 15 cm (d)	Reduction of corrosion currents and corrosion rates; enhancement of polarizationresistances	[100]
PU	Metal Coating	Emulsion polymerization	Particle	35–95	Cyclohexane(s), 45 °C (T), 8 h (t)	25% of the material used for same application	[101]
PAN	Reinforced Adhesive	Electrospinning	Fiber	362 ± 87	THF (s), 10 wt % (c), 16 kV (V), 1 mL/h (f.r.), 15 cm (d)	Enhancement for the mechanical property	[102]
PA66	Flame Retardant	Electrospinning	Fiber	225–425	Formic acid (s), 15–20 wt % (c), 30 kV (V), 0.5 mL/h (f.r.), 8 cm (d)	Mechanical properties and flame-retardancy improved	[103]

c—concentration; d—distance; f.r. —feed rate; s—solvent; V—voltage.

## References

[1] Needham R.B., Doe P.H. (1987). Polymer flooding review. J. Pet. Technol..

[2] Cheung H.Y., Lau K.T., Lu T.P., Hui D. (2007). A critical review on polymer-based bio-engineered materials for scaffold development. Compos. Part B.

[3] Shrive N.G. (2006). The use of fibre reinforced polymers to improve seismic resistance of masonry. Constr. Build. Mater..

[4] Halliwell S.M. (2002). Polymers in Building and Construction.

[5] Herrmann A.S., Nickel J., Riedel U. (1998). Construction materials based upon biologically renewable resources-from components to finished parts. Polym. Degrad. Stab..

[6] Feldman D. (2014). Polymer nanocomposites in building, construction. J. Macromol. Sci. Part A Pure Appl. Chem..

[7] Rana1 A.K., Rana S.B., Kumari A., Kiran V. (2009). Significance of nanotechnology in construction engineering. Int. J. Recent Trends Eng..

[8] Torgala F.P., Jalali S. (2011). Nanotechnology: Advantages and drawbacks in the field of construction and building materials. Constr. Build. Mater..

[9] Raoa J.P., Geckeler K.E. (2011). Polymer nanoparticles: Preparation techniques and size-control parameters. Prog. Polym. Sci..

[10] Zhao B., Deng J. (2016). Emulsion polymerization of acetylenics for constructing optically active helical polymer nanoparticles. Polym. Rev..

[11] Huang Z.M., Zhang Y.Z., Kotakic M., Ramakrishna S. (2003). A review on polymer nanofibers by electrospinning and their applications in nanocomposites. Compos. Sci. Technol..

[12] Arinstein A., Zussman E. (2011). Electrospun polymer nanofibers: Mechanical and thermodynamic perspectives. J. Polym. Sci. Part B Polym. Phys..

[13] Reis C.P., Neufeld R.J., Ribeiro A.J., Veiga F. (2006). Nanoencapsulation I. Methods for preparation of drug-loaded polymeric nanoparticles. Nanomed. Nanotechnol. Biol. Med..

[14] Jang J.S., Oh J.H. (2002). Novel crystalline supramolecular assemblies of amorphous polypyrrole nanoparticles through surfactant templating. Chem. Commun..

[15] Zhang Q., Chuang K.T. (2001). Adsorption of organic pollutants from effluents of a kraft pulp mill on activated carbon and polymer resin. Adv. Environ. Res..

[16] Pal S.L., Jana U., Manna P.K., Mohanta G.P., Manavalan R. (2011). Nanoparticle: An overview of preparation and characterization. J. Appl. Pharm. Sci..

[17] Julienne V.M.C., Benoit J.P. (1996). Preparation, purification and morphology of polymeric nanoparticles as drug carriers. Pharm. Acta Helv..

[18] Crucho C.I.C., Barros M.T. (2015). Formulation of functionalized PLGA polymeric nanoparticles for targeted drug delivery. Polymer.

[19] Bilati1 U., Allemann E., Doelker E. (2005). Development of a nanoprecipitation method intended for the entrapment of hydrophilic drugs into nanoparticles. Eur. J. Pharm. Sci..

[20] Song X. (2008). PLGA nanoparticles simultaneously loaded with vincristine sulfate and verapamil hydrochloride: Systematic study of particle size and drug entrapment efficiency. Int. J. Pharm..

[21] Allemann E., Gurny R., Doelker E. (1992). Preparation of aqueous polymeric nanodispersions by a reversible salting-out process: Influence of process parameters on particle size. Int. J. Pharm..

[22] Jeong Y.I., Cho C.S., Kim S.H., Ko K.S., Kim S.I., Shim Y.H., Nah J.W. (2001). Preparation of poly(dl-lactide-*co*-glycolide) nanoparticles without surfactant. J. Appl. Polym. Sci..

[23] Thickett S.C., Gilbert R.G. (2007). Emulsion polymerization: State of the art in kinetics and mechanisms. Polymer.

[24] Soppimath K.S., Aminabhavi T.M., Kulkarni A.R., Rudzinski W.E. (2001). Biodegradable polymeric nanoparticles as drug delivery devices. J. Control. Release.

[25] Yuan L., Wang Y., Pan M., Rempela G.L., Pan Q. (2013). Synthesis of poly(methyl methacrylate) nanoparticles via differential microemulsion polymerization. Eur. Polym. J..

[26] Wu M., Dellacherie E., Durand A., Marie E. (2009). Poly(*N*-butyl cyanoacrylate) nanoparticles via miniemulsion polymerization (1): Dextranbased surfactants. Colloid Surf. B.

[27] Hirech K., Payan S., Carnelle G., Brujes L., Legrand J. (2003). Microencapsulation of an insecticide by interfacial polymerization. Powder Technol..

[28] Scott C., Wu D., Ho C.C., Co C.C. (2005). Liquid-core capsules via interfacial polymerization: A free-radical analogy of the nylon rope trick. J. Am. Chem. Soc..

[29] Desgouilles S., Vauthier C., Bazile D., Vacus J., Grossiord J.L., Veillard M., Couvreur P. (2003). The design of nanoparticles obtained by solvent evaporation: A comprehensive study. Langmuir.

[30] Higuchi T., Yabu H., Shimomura M. (2006). Simple preparation of hemispherical polystyrene particles. Colloid Surf. A.

[31] Fessi H., Puisieux F., Devissaguet J.P., Ammoury N., Benita S. (1989). Nanocapsule formation by interfacial polymer deposition following solvent displacement. Int. J. Pharm..

[32] Ganachaud F., Katz J.L. (2005). Nanoparticles and nanocapsules created using the ouzo effect: Spontaneous emulsification as an alternative to ultrasonic and high-shear devices. ChemPhysChem.

[33] Noh M., Lee D. (1999). Synthesis and characterization of PS-clay nanocomposite by emulsion polymerization. Polym. Bull..

[34] Lu S., Qu R., Forcada J. (2009). Preparation of magnetic polymeric composite nanoparticles by seeded emulsion polymerization. Mater. Lett..

[35] Baji A., Mai Y.W., Wong S.C., Abtahi M., Chen P. (2010). Electronspinning of polymer nanofibers: Effects on oriented morphology, structures and tensile properties. Compos. Sci. Technol..

[36] Persano L., Camposeo A., Tekmen C., Pisignano D. (2013). Industrial upscaling of electrospinning and applications of polymer nanofibers: A Review. Macromol. Mater. Eng..

[37] Ondarcuhu T., Joachim C. (1998). Drawing a single nanofibre over hundreds of microns. Eur. Phys. Lett..

[38] Martin C.R. (1996). Membrane-based synthesis of nanomaterials. Chem. Mater..

[39] Nam Y.S., Park T.G. (1999). Biodegradable polymeric microcellular foams by modifed thermally induced phase separation method. Biomaterials.

[40] Liu G.J., Ding J.F., Qiao L.J., Guo A., Dymov B.P., Gleeson J.T. (1999). Polystyrene-*block*-poly(2-cinnamoylethyl methacrylate) nanofibers: Preparation, characterization, and liquid crystalline properties. Chem. A Eur. J..

[41] Frenot A., Chronakis I.S. (2003). Polymer nanofibers assembled by electrospinning. Curr. Opin. Colloid Interface Sci..

[42] Megelski S., Stephens J.S., Rabolt J.F., Bruce C.D. (2002). Micro- and nanostructured surface morphology on electrospun polymer fibers. Macromolecules.

[43] Lee K.H., Kim H.Y., La Y.M., Lee D.R., Sung N.H. (2002). Influence of a mixing solvent with tetrahydrofuran and *N*,*N*-dimethylformamide on electrospun poly(vinylchloride) nonwoven mats. J. Polym. Sci Part B Polym. Phys..

[44] Serkis-Rodzeń M., Spírková M., Matejícek P., Stepánek M. (2017). Formation of linear and crosslinked polyurethane nanoparticles that self-assemble differently in acetone and in water. Prog. Org. Coat..

[45] Fu H., Gao H., Wu G., Wang Y., Fan Y., Ma J. (2011). Preparation and tunable temperature sensitivity of biodegradable polyurethane nanoassemblies from diisocyanate and poly(ethylene glycol). Soft Matter.

[46] Wang A., Gao H., Sun Y., Sun Y., Yang Y., Wu G., Wang Y., Fan Y., Ma J. (2013). Temperature- and pH-responsive nanoparticles of biocompatible polyurethanes for doxorubicin delivery. Int. J. Pharm..

[47] Ramos B.J.Z., Sennab E.L., Soldia V., Borsalic R., Cloutetc E., Cramail H. (2006). Polyurethane nanoparticles from a natural polyol via miniemulsion technique. Polymer.

[48] Shendi H.K., Omrani I., Ahmadi A., Farhadian A., Babanejad N., Nabid M.R. (2017). Synthesis and characterization of a novel internal emulsifier derived from sunflower oil for the preparation of waterborne polyurethane and their application in coatings. Prog. Org. Coat..

[49] Zhuo H., Hu J., Chen S., Yeung L. (2008). Preparation of polyurethane nanofibers by electrospinning. J. Appl. Polym. Sci..

[50] Hu J., Wang X., Ding B., Lin J., Yu J., Sun G. (2011). One-step electro-spinning/netting technique for controllably preparing polyurethane nano-fiber/net. Macromol. Rapid Commun..

[51] Lev J., Holba M., Došek M., Kalhotka L., Mikulǎ P., Kimmer D. (2014). A novel electrospun polyurethane nanofibre membrane—Production parameters and suitability for wastewater (WW) treatment. Water Sci. Technol..

[52] Lev J., Holba M., Kalhotk L., Mikula P., Kimmer D. (2012). Improvements in the structure of electrospun polyurethane nanofibrous materials used for bacterial removal from wastewater. Inter. J. Theor. Appl. Nanotechnol..

[53] Guan K., Zhuang X., Yan G., Cheng B. (2011). Fabrication and properties of polyurethane nanofibers nonwoven by solution blowing. Adv. Mater. Res..

[54] Polat Y., Pampal E.S., Stojanovska E., Simsek R., Hassanin A., Kilic A., Demir A., Yilmaz S. (2016). Solution blowing of thermoplastic polyurethane nanofibers: A facile method to produce flexible porous materials. J. Appl. Polym. Sci..

[55] Ernawati L., Balgis R., Ogi T., Okuyama K., Takada T. (2017). Role of acetone in the formation of highly dispersed cationic polystyrene nanoparticles. Chem. Process Eng..

[56] Liu X., Lu Y., Luo G. (2017). Continuous flow synthesis of polystyrene nanoparticles via emulsion polymerization stabilized by a mixed nonionic and anionic emulsifier. Ind. Eng. Chem. Res..

[57] Asran A.S., Seydewitz V., Michler G.H. (2012). Micromechanical properties and ductile behavior of electrospun polystyrene nanofibers. J. Appl. Polym. Sci..

[58] Hashemifard N., Shariati S. (2016). Electrospun polystyrene nanofiber as an adsorbent for solid-phase extraction of disulfine blue from aqueous samples. Arab. J. Sci. Eng..

[59] Liu F., Song D., Huang X., Xu H. (2016). Electrospun polystyrene nanofibers as a novel adsorbent to transfer an organic phase from an aqueous phase. J. Sep. Sci..

[60] Lee M.W., An S., Latthe S.S., Lee C., Hong S., Yoon S.S. (2013). Electrospun polystyrene nanofiber membrane with superhydrophobicity and superoleophilicity for selective separation of water and low viscous oil. ACS Appl. Mater. Interfaces.

[61] Li H., Zhang Q., Guo N., Zhu A., Liu Q. (2015). Ultrafine polystyrene nanofibers and its application in nanofibrous membranes. Chem. Eng. J..

[62] Landfester K., Antonietti M. (2000). The polymerization of acrylonitrile in miniemulsions: “Crumpled latex particles” or polymer nanocrystals. Macromol. Rapid Commun..

[63] Jeon H.J., You Y., Yoon M.J., Youk J.H. (2011). Preparation of polyacrylonitrile nanoparticles via dispersion polymerization of acrylonitrile using a poly(*N*-vinyl pyrrolidone)-cobalt complex in an aqueous system. Polymer.

[64] Zhang Y., Zhuang X., Gu W., Zhao J. (2015). Synthesis of polyacrylonitrile nanoparticles at high monomer concentrations by AIBN-initiated semi-continuous emulsion polymerization method. Eur. Polym. J..

[65] Lee I., Kim S., Kim S., Jang Y., Jan J. (2014). Highly fluorescent amidine/schiff base dual-modified polyacrylonitrile nanoparticles for selective and sensitive detection of copper ions in living cells. ACS Appl. Mater. Interfaces.

[66] Zhang J., Lu Z., Wu M., Wu Q., Yang J. (2015). Large-scale synthesis and characterization of magnetic poly(acrylic acid) nanogels via miniemulsion polymerization. RSC Adv..

[67] Youm J.S., Kim J.H., Kim C.H., Kim J.C., Kim Y.A., Yang K.S. (2016). Densifying and strengthening of electrospun polyacrylonitrile-based nanofibers by uniaxial two-step stretching. J. Appl. Polym. Sci..

[68] Kim H.Y., Kim B., Lee B.C., Koo1 Y.H., Baeck S.H., Shim S.E. (2015). Structure evolution of electrospun polyacrylonitrile nanofibers by electron beam irradiation. Fibers Polym..

[69] Wang W., Liu Y., Wang Y., Chen H., Bai L. (2017). A novel and convenient preparation of antibacterial polyacrylonitrile nanofibers via post-modification using nitrile click chemistry and electrospinning. Chem. Pap..

[70] Almasiana A., Fardb C., Gashtic M.P., Mirjalilic M., Mokhtari Z., Shourijeh M. (2016). Surface modification of electrospun PAN nanofibers by amine compounds for adsorption of anionic dyes. Desalin. Water Treat..

[71] Dilpazir S., Usman M., Rasulband S., Arshad S.N. (2016). A simple UV-Ozone surface treatment to enhance photocatalytic performance of TiO_2_ loaded polymer nanofiber membranes. RSC Adv..

[72] Makaremi M., Lim C.X., Pasbakhsh P., Lee S.M., Goh K.L., Chang H., Chan E.S. (2016). Electrospun functionalized polyacrylonitrile–chitosan Bi-layer membranes for water filtration applications. RSC Adv..

[73] Elkhaldi R.M., Guclu S., Koyuncu I. (2016). Enhancement of mechanical and physical properties of electrospun PAN nanofiber membranes using PVDF particles. Desalin. Water Treat..

[74] Jang J., Bae J., Park E. (2006). Polyacrylonitrile nanofibers: Formation mechanism and applications as a photoluminescent material and carbon-nanofiber precursor. Adv. Funct. Mater..

[75] Vatani Z., Eisazadeh H. (2013). Coating of poly(vinyl chloride) nanoparticles with a conductive polyaniline in the presence of various surfactants. J. Vinyl Addit. Technol..

[76] Phatcharasit K., Taweepreda W., Boonkerd K., Kim H.K. (2013). Preparation and properties of electrospun PVC nanofiber. Adv. Mater. Res..

[77] Jiang T., Kang W., Cheng B. (2012). Preparation and characterization of electrospun CPVC nanofiber nonwovens. Adv. Mater. Res..

[78] Krupa A., Sobczyk A.T., Jaworek A. (2014). Surface properties of plasma-modifed poly(vinylidene fluoride) and poly(vinyl chloride) nanofbres. Fibers Text. East. Eur..

[79] Guo N., Zhang Q., Li H., Wu X., Liu Q., Zhu A. (2014). Facile fabrication, structure, and applications of polyvinyl chloride mesoporous membranes. Ind. Eng. Chem. Res..

[80] Tauer K., Qoebel K.H., Kosmella S., Stahler K., Neelsen J. (1990). Emulsion polymerization in the presence of polymerizible emulsifiers and surface active initiators. Macromol. Symp..

[81] Wu H., Khalid B., Bai X., Wei H., Huang Y., Cui Y. (2017). Direct blow-spinning of nanofibers on window screen for highly efficient PM_2.5_ removal. Nano Lett..

[82] Jing L., Shim K., Toe C.Y., Fang T., Zhao C., Amal R., Sun K., Kim J.H., Ng Y.H. (2016). Electrospun polyacrylonitrile-ionic liquid nanofibers for superior PM_2.5_ capture capacity. ACS Appl. Mater. Interfaces.

[83] Zhang R., Liu C., Hsu P.-C., Zhang C., Liu N., Zhang J., Lee H.R., Lu Y., Qiu Y., Chu S. (2016). Nanofiber air filters with high-temperature stability for efficient PM_2.5_ removal from the pollution sources. Nano Lett..

[84] Xu J., Liu C., Hsu P.C., Liu K., Zhang R., Liu Y., Cui Y. (2016). Roll-to-Roll transfer of electrospun nanofiber film for high-efficiency transparent air filter. Nano Lett..

[85] Li Q., Xu Y., Wei H., Wang X. (2016). Electrospun polycarbonate nanofibrous membrane for high efficiency particulate matter filtration. RSC Adv..

[86] Zhang S., Liu H., Yin X., Yu J., Ding B. (2016). Anti-deformed polyacrylonitrile/polysulfone composite membrane with binary structures for effective air filtration. ACS Appl. Mater. Interfaces.

[87] Wang S., Zhao X., Yin X., Yu J., Ding B. (2016). Electret polyvinylidene fluoride nanofibers hybridized by polytetrafluoroethylene nanoparticles for high-efficiency air filtration. ACS Appl. Mater. Interfaces.

[88] Su J., Yang G., Cheng C., Huang C., Xu H., Ke Q. (2017). Hierarchically structured TiO_2_/PAN nanofibrous membranes for high-efficiency air filtration and toluene degradation. J. Colloid Interface Sci..

[89] Sarı A., Alkan C., Doguscu D.K., Kızıl C. (2015). Micro/nano encapsulated *n*-tetracosane and *n*-octadecane eutectic mixture with polystyrene shell for low-temperature latent heat thermal energy storage applications. Sol. Energy.

[90] Fang Y., Kuang S., Gao X., Zhang Z. (2008). Preparation and characterization of novel nanoencapsulated phase change materials. Energy Convers. Manag..

[91] Cho W., Kook J.W., Lee S.M., Koh W.G., Kim J.H. (2016). Modification of heat storage ability and adhesive properties of core/shell structured phase change material nanocapsules. Macromol. Res..

[92] Lu P., Chen W., Zhu M., Murray S. (2017). Embedding lauric acid into polystyrene nanofibers to make high-capacity membranes for efficient thermal energy storage. ACS Sustain. Chem. Eng..

[93] Mu S., Guo J., Zhang B., Qia S., Yang L., Wang D., Zhang S., Yu Y. (2015). On preparation and characterization of the phase change nanofibers from the copolymer of poly(styrene-*co*-acrylonitrile) and lauric acid. J. Macromol. Sci. Part A Pure Appl. Chem..

[94] Ke H. (2016). Electrospun methyl stearate/PET form-stable phase change composite nanofbres for storage and retrieval of thermal energy. Mater. Res. Innov..

[95] Kook J.W., Cho W., Koh W.G., Cheong I.W., Kim J.H. (2015). Preparation and characterization of octadecane/polyurea nanocapsule-embedded poly(ethylene oxide) nanofibers. J. Appl. Polym. Sci..

[96] Rabbi A., Bahrambeygi H., Nasouri K., Shoushtari A.M., Babaei M.R. (2014). Manufacturing of PAN or PU nanofiber layers/PET nonwoven composite as highly effective sound absorbers. Adv. Polym. Technol..

[97] Bahrambeygi H., Sabetzadeh N., Rabbi A., Nasouri K., Shoushtari A.M. (2013). Nanofibers (PU and PAN) and nanoparticles (Nanoclay and MWNTs) simultaneous effects on polyurethane foam sound absorption. J. Polym. Res..

[98] Xiang H., Tan S., Yu X., Long Y., Zhang X. (2011). Sound absorption behavior of electrospun polyacrylonitrile nanofibrous membranes. Chin. J. Polym. Sci..

[99] Wu C.M., Chou M.H. (2016). Polymorphism, piezoelectricity and sound absorption of electrospun PVDF membranes with and without carbon nanotubes. Compos. Sci. Technol..

[100] Gaballah S., Shehata N., Shaaban M., Nosier S., Hefnawy A., Hamed A., Samir E. (2017). Corrosion inhibition of aluminum in Hydrochloric acid solution using ceria doped polyvinyl chloride nanofiber. Int. J. Electrochem. Sci..

[101] Müller K., Klapper M., Müllen K. (2007). Preparation of high molecular weight polyurethane particles by nonaqueous emulsion polyaddition. Colloid Polym. Sci..

[102] Razavi S.M.J., Neisiany R.E., Ayatollahi M.R., Ramakrishna S., Khorasani S.N., Berto F. (2017). Fracture assessment of polyacrylonitrile nanofiber-reinforced epoxy adhesive. Theor. Appl. Fract. Mech..

[103] Xiao L., Xu L., Yang Y., Zhang S., Huang Y., Bielawski C.W., Geng J. (2017). Core-shell structured polyamide 66 nanofibers with enhanced flame retardancy. ACS Omega.

